# *Dientamoeba fragilis* in Gaza Strip: a Neglected Protozoan Parasite

**Published:** 2013

**Authors:** Adnan I. Al-Hindi, Basma M. Abu Shammala

**Affiliations:** Department of Biology, Faculty of Science, the Islamic University of Gaza, P.O.Box 108, Gaza, Palestine

**Keywords:** *Dientamoeba fragilis*, Prevalence, Diagnosis, Gaza

## Abstract

**Background:**

The aim of this study was to detect *Dientamoeba fragilis* by iron haematoxylin stain, as well as its prevalence, and association between *D. fragilis* infection and diarrhoea among patients attending Al-Nuseirate Refugee Camp Clinic, Gaza Strip.

**Methods:**

A cross-sectional study was conducted among 319 children and adults with age ranges from (1 to 75) years old, attending Al-Nussirat Clinic, and who were complaining from clinical symptoms, like diarrhoea and abdominal pain.

**Results:**

28 individuals were infected with *D. fragilis* with a prevalence of 8.8%. The detection of 28 cases infected with *D. fragilis* was proved using iron haematoxylin stain, but no case was detected by direct smear or formal-ether sedimentation technique. The most frequent symptoms were abdominal pain (96.4%) and diarrhoea (71.4%) in patients with diantamoebiasis and this was statistically significant (*P*= 0.03). Co-infection between *D. fragilis* and *Entamoeba histolytica/dispar* was 50% and between *D. fragilis* and *Giardia lamblia* was 7.1%.

**Conclusion:**

*D. fragilis* was present in the patients stool samples and was detected and proved using iron haematoxylin stain.

## Introduction

Studies have proved that there are many places in Gaza strip especially in refugee camps which has a considerable prevalence of intestinal parasites. This leads to a great dangerous on the health of those people especially children (1–4). Diagnosis of intestinal parasites in Gaza is still a problem, where in hospitals or private laboratories, the direct smear method is the only common method used. The use of staining, antigen detection, serological methods, DNA-based methods are unavailable in routine testing, but have been applied in research cases (5). Protozoa such as *Dientamoeba fragilis* remain neglected.

*Dientamoeba fragilis* is a pathogenic human intestinal protozoan parasite first described in the scientific literature in 1918 by Jeeps and Dobell. Because of the lack of a cyst stage, successful diagnosis of *D. fragilis* is closely associated with the use of permanent stains of faecal smears (6). The pathogenicity of *D. fragilis* has been debated for many years. Today clinical symptoms such as abdominal pain, diarrhoea, anorexia, nausea, vomiting and flatulence, which usually disappear with the elimination of the parasite, are reported to be associated with *D. fragilis* (7, 8). It has a worldwide distribution, with a prevalence ranging from 1.2% to 52.2% (9, 10).

The aim of this study was to detect *D. fragilis* by iron haematoxylin stain, among patients attending Al-Nuseirate Refugee Camp Clinic; determination of the prevalence of *D. fragilis* among those patients and association between *D. fragilis* infection and diarrhoea.

## Materials and Methods

### Description of the Study area

Nuseirate Refugee Camp (NRC) was established in 1949 on a 0.588 km^2^ site, is located 8 km south of Gaza City and its population was 44.425 in 1997, 64.233 in 2002 and estimated to be 83.033 people in 2010. Water supply to the NRC is mainly from wells in which chloride concentration reaches 1200 mg/l in summer. UNRWA's health centre, which was built in 1963, provides outpatient medical care and other specialized health services (11, 12).

NRC includes eight Blocks named Block B, D, F, G, H, J, and L and M. many of these Blocks have a poor sewage network; sewage and wastewater flow in open channels along rods and pathways, and through agricultural lands towards Wadi Gaza, thus posing serious environmental health hazard (13).

### Source of specimens

This study was carried out in Gaza Strip, where 319 stool specimens were collected from children and adults with age ranges from 1 to 75 years old, attending Al-Nussirat clinic, and who were complaining from clinical symptoms, like diarrhoea and abdominal pain. The selection of individuals was based on regular times starting from 8 a.m to 12:30 p.m for three days weekly. A part of each stool specimen was preserved with sodium acetate acetic acid formalin (SAF) for staining purposes. All stool specimens were examined previously by direct smear microscopy (Saline and Lugoe's iodine), and staining by iron haematoxylin and found to be positive for the trophozoite of *Dientamoeba fragilis*. All stool specimens were also examined macroscopically for the presence of blood, and staining by iron haematoxylin and found to be positive for the trophozoite of *Dientamoeba fragilis* mucus and for consistency.

### Formol-ether sedimentation technique

This concentration method was used to obtain sediment to detect any protozoa according WHO (14).

### Staining by iron haematoxylin

The staining by iron haematoxylin was carried out according to the staining protocol of WHO (14).

### Ethical considerations

Ethical approval was obtained from the director of the Health Department in the UNRWA, the director of the Al-Nuseirate laboratory and Helsinki Committee in Gaza Strip to facilitate the collection of stool sample from patients attending the clinic. All patients were informed verbally about the purpose of the study.

### Statistical analysis

The collected data were analyzed by using SPSS (Statistical Package for Social Studies version 12). Analysis of variables, frequency tables, cross tabulation, T-test, and graphs were carried out. Excel programme was used for the graphics.

## Results

It was found that 28/319 of these individuals were infected with *D. fragilis* with a prevalence of (8.8%). The study sample compromised 164 males (51.4%) and 155 females (48.6%) and who reside in one of three localities, city, village or refugee camp.

The most common character for stool consistency was the softness with a prevalence of 63.3%. Totally, 97% of patients were symptomatic, 93.7% had abdominal pain and 53.9% had diarrhoea. No available information on combinations of symptoms was recorded.

**Fig. 1:**
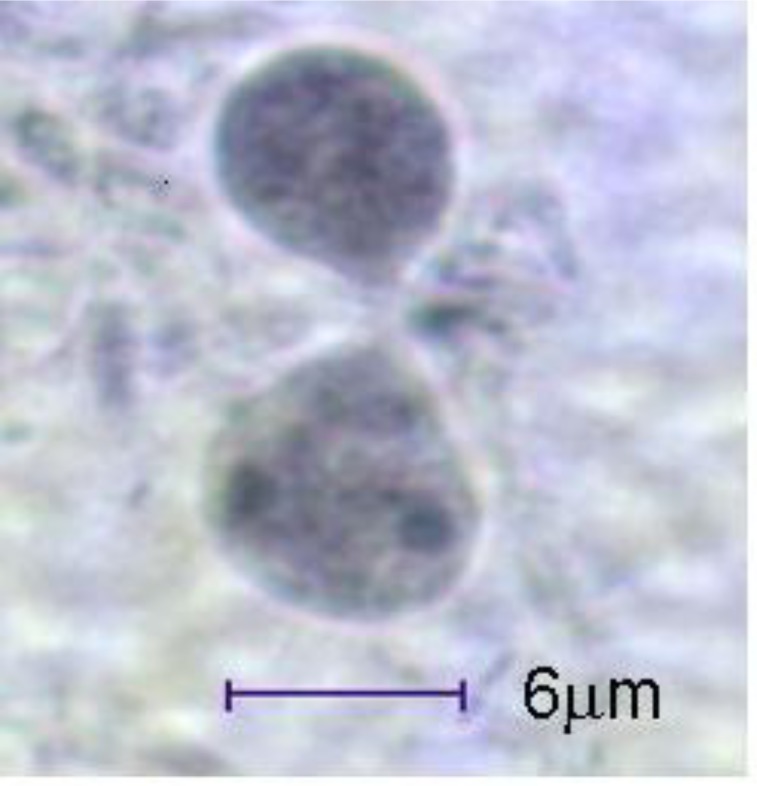
*Dientamoeba fragilis* trophozoite stained by iron haematoxylin stain (Photo by Adnan Al-Hindi, 2005)

Stool examination results proved that the prevalence of intestinal parasites was 18.2%. There were difference in the ratio between the detected intestinal parasites from our results and from the questionnaire, for example the prevalence of *E. histolytica* from our results was 14.7% but it was 5.0% from questionnaire (Tables 1 and 2). The detected trophozoite of *Dientamoeba fragilis* was stained by iron haematoxylin as shown in Fig. 1.

**Table 1:** The detected types of intestinal parasites

Type of parasite	The detected parasites as shown by wet mount using saline		The detected parasites as shown from questionnaire	

	No.	%	No.	%
*Entamoeba histolytica/dispar*	47	14.7	16	5.0
*Giardia lamblia*	18	5.6	19	6.0
*Dientamoeba fragilis*	28	8.8	-	-
*Ascaris lumbricoides*	2	0.6	6	1.9
*Enterobius vermicularis*	-	-	32	10.0
*Hymenolypes nana*	1	0.3	-	-
*Trichomonas hominis*	3	0.9	-	-

**Table 2:** Consistency of stool sample with *Dientamoeba fragilis* infection (n = 319)

Symptoms	Positive for *Dientamoeba fragilis*		Negative for *Dientamoeba fragilis*	

	No.	%	No.	%
Formed (n = 4)	0	0	4	(100)
Soft (n = 202)	11	(5.4)	191	(94.6)
Loose (n = 32)	3	(9.4)	29	(90.6)
Watery (n = 19)	2	(10.5)	17	(89.5)
Mucoid (n = 38)	6	(15.8)	32	(84.2)
Mucoid and Bloody (n = 17)	4	(23.5)	13	(76.5)
Watery and Mucoid (n = 7)	2	(28.6)	5	(71.4)

The patients with *D. fragilis* infection had high prevalence of diarrhoea (71.4%) compared to patients with no diarrhoea (28.6%) (*P*= 0.03). 96.4% of patients infected with *D. fragilis* had abdominal pain, and 17.9% had bloody stool. Patients who did not have *D. fragilis* commonly had the same symptoms (Table 3).

**Table 3:** Distribution of *Dientamoeba fragilis* with self-reported symptoms (n = 319)

Symptom	Positive for *Dientamoeba fragilis*		Negative for *Dientamoeba fragilis*		*P Value
	No.	%	No.	%	
With symptoms(n = 312)	28	(100)	284	(97.6)	
Without symptoms (n = 7)	0		7	(2.4)	> 0.05
Had constipation (n = 94)	5	(17.9)	89	(30.6)	
Had no constipation (n = 225)	23	(82.1)	202	(69.4)	> 0.05
Had abdominal pain (n—299)	27	(96.4)	272	(93.5)	
Had no abdominal pain (n = 20)	1	(3.6)	19	(6.5)	> 0.05
Had bloody stool (n = 46)	5	(17.9)	41	(14.1)	
Had no bloody stool (n—273)	23	(82.1)	250	(85.9)	> 0.05
Had wormed expelled (n = 80)	5	(17.9)	75	(25.8)	
Had no wormed expelled(n = 239)	23	(82.1)	216	(74.2)	>0.05
Had itching (n—76)	5	(17.9)	71	(24.4)	
Had no itching (n—243)	23	(82.1)	220	(75.6)	>0.05
Had diarrhea (n—172)	20	(71.4)	152	(52.2)	
Had no diarrhea (n = 147)	8	(28.6)	139	(47.8)	0.03

* P< 0.05 is significant

The distribution of *D. fragilis* in the case of opened sewer was more than from closed sewer. Homes with prevalence of garbage had high prevalence with *D. fragilis* (18.9%) compared to homes without garbage (7.4%). These differences were not statistically significant (Table 4).

**Table 4:** Distribution of *Dientamoeba fragilis due* to different variables

Parameter		With *Dientamoeba fragilis*		Without *Dientamoeba fragilis*		(χ^2^ *P* -Value)

		No.	%	No.	%	
**Age groups**						
< 5 years old	(n—53)	6	(11.3)	47	(88.7)	
6-12 years old	(n = 109)	10	(9.2)	99	(90.8)	
13-19 years old	(n—53)	3	(5.7)	50	(94.3)	(3.064, > 0.05)
20-26years old	(n—26)	4	(5.9)	22	(84.6)	
27-33 years old	(n—17)	1	(15.4)	16	(94.1)	
> 34 years old	(n—61)	4	(6.6)	57	(93.4)	
**Sex**				149	(90.9)	(0.057,> 0.05)
Males	(n—164)	15	(9.1)	142	(91.6)	
Females	(n—155)	13	(8.4)			
**Sewers type**						(0.35, > 0.05)
Closed sewage	(n—272)	21	(7.7)	251	(92.3)	
Opened sewage	(n—47)	7	(14.9)	40	(85.1)	
**Prevalence of garbage**						
There is garbage	(n—37)	7	(18.9)	30	(81.1)	
There is no garbage	(n—282)	21	(7.4)	261	(92.6)	> 0.05
**Father occupation**						
Employer	(n = 73)	6	(8.2)	67	(91.8)	
Laborer	(n—108)	10	(9.3)	98	(90.7)	> 0.05
Un-employment	(n = 135)	12	(8.9)	123	(91.1)	
Dead (n—3)		0		3	(100.0)	

The highest prevalence with *D. fragilis* (15.4%) was in the age group (20-26 years old) (Table 4). The prevalence among males was 9.1% and 8.4% among females, but no significant relationship was found. Fourteen samples of *E. histolytica/dispar* were associated with *D. fragilis out of* 28 (50%). Information on symptoms or combination of symptoms is presented in Table 5.

**Table 5:** Double infection with Giardia lamblia or Entamoeba histolytica/dispar

*Dientamoeba fragilis*	Infection with *Giardia lamblia*	Infection with *Entamoeba histolytica/dispar*
2	−	−
7	−	−
19	−	+
25	+	−
35	−	−
41	−	−
50	−	−
57	−	−
62	−	+
65	−	−
68	+	−
82	−	−
97	−	+
106	−	+
109	−	+
111	−	+
119	−	−
120	−	+
138	−	−
148	−	+
155	−	+
172	−	+
184	−	+
186	−	+
248	−	+
257	−	+
306	−	−
314	−	−
	**2/28 (7.1%)**	**14/28 (50%)**

## Discussion

Traditional diagnosis of intestinal parasites using direct smear microscopy remains the recognized method in Gaza. This was the first study regarding the occurrence of *D. fragilis* in Gaza. The prevalence of *D. fragilis* was 8.8%. Other researchers found results of 8.9% among patients in Egypt (15), in Libya 2% (16), in Italy 3.7% (17), and in Turkey 8.8% (18).

The diagnosis of dientamoebiasis depends on the demonstration of the parasite, which typically has two (or sometimes one) nuclei, each containing 4-6 granules. A permanent stain such as trichrome or iron haematoxylin is obligatory in the diagnosis of *D. fragilis* and experience and attention are also required, even when stains are performed (19, 20).

In the present study, the detection of 28 cases infected with *D. fragilis* was proved using iron haematoxylin stain, but no case was detected by direct smear or formal- ether sedimentation technique. Moreover, during the search in the local literature and the annual reports of Ministry of Health no mention for this parasite was made. This may be due to the lack of knowledge about this protozoan and the inappropriate laboratory techniques, which are confined to direct smear in the local hospitals and private laboratories in Gaza.

To date, the only reliable method for the laboratory diagnosis of *D. fragilis* is microscopic examination of stained smears of preserved stool specimens. Laboratories which do not use this procedure would almost certainly miss the diagnosis (21).

After using a suitable faecal stain as a part of routine methodology Windsor et al., (22) found *D. fragilis* to be the most common enteropathogen (5.1%) in the Sultanate of Oman. Five percent has been reported recently among American soldiers stationed in Egypt (23).

The present findings indicated that watery and mucoid stool samples had high prevalence of *D. fragilis* (28.6%), although numbers were small. Uninfected individuals showed a proportion of 84.2% with mucoid stool and 76.5% showed mucoid and bloody stool. This could be attributed to other causes rather than *Dientamoeba* like *Shigella* or rotaviruses.

In the present study, regarding age the infection was found to be not statistically significant (*P*>0.05). Children under five years old had a prevalence of 11.3% while the age group (20–26) years old had 15.4%. This finding is different from the findings by Girginkardesler et al., (18) who reported that *D. fragilis* infection was higher among children than adult.

The association between *D. fragilis* and diarrhoea may represent evidence for the pathogenicity of *D. fragilis*. Numerous studies have shown that a large proportion of patients with *D. fragilis* infection have other enteric protozoa present, all of which are transmitted via the faecal-oral rout. For example, Stark et al., (24) found that 40% of patients with dientamoebia-sis were co-infected with other parasites. In addition, Windsor et al., (22) found that 54% of patients with *D. fragilis* had other parasites or enteropathogens present. In the present study co-infection between *D. fragilis* and *E. histolytica/dispar* and *G. lamblia* were found, where co-infection between *D. fragilis* and *E. histolytica/dispar* was 50% and between *D. fragilis* and *G. lamblia* was 7.1%.

## Limitations

As with other studies, resources did not permit the collection of more than one stool from each patient and the true extent of parasites like *D. fragilis* could be underestimated.

## Conclusions

1. Dientamoebiasis should be further investigated as part of a proposed strategy to improve the diagnosis of parasites in Gaza.
2. The degree to which diarrhoeas require investigation should be discussed in MOH and associated medical laboratories.
3. The role of iron haematoxylin and other staining methods requires clarification.
